# Assessing the Performance Capabilities of LRE-Based Assays for Absolute Quantitative Real-Time PCR

**DOI:** 10.1371/journal.pone.0009731

**Published:** 2010-03-17

**Authors:** Robert G. Rutledge, Don Stewart

**Affiliations:** Canadian Forest Service, Natural Resources Canada, Quebec, Quebec, Canada; Texas A&M University, United States of America

## Abstract

**Background:**

Linear regression of efficiency or LRE introduced a new paradigm for conducting absolute quantification, which does not require standard curves, can generate absolute accuracies of ±25% and has single molecule sensitivity. Derived from adapting the classic Boltzmann sigmoidal function to PCR, target quantity is calculated directly from the fluorescence readings within the central region of an amplification profile, generating 4–8 determinations from each amplification reaction.

**Findings:**

Based on generating a linear representation of PCR amplification, the highly visual nature of LRE analysis is illustrated by varying reaction volume and amplification efficiency, which also demonstrates how LRE can be used to model PCR. Examining the dynamic range of LRE further demonstrates that quantitative accuracy can be maintained down to a single target molecule, and that target quantification below ten molecules conforms to that predicted by Poisson distribution. Essential to the universality of optical calibration, the fluorescence intensity generated by SYBR Green I (FU/bp) is shown to be independent of GC content and amplicon size, further verifying that absolute scale can be established using a single quantitative standard. Two high-performance lambda amplicons are also introduced that in addition to producing highly precise optical calibrations, can be used as benchmarks for performance testing. The utility of limiting dilution assay for conducting platform-independent absolute quantification is also discussed, along with the utility of defining assay performance in terms of absolute accuracy.

**Conclusions:**

Founded on the ability to exploit lambda gDNA as a universal quantitative standard, LRE provides the ability to conduct absolute quantification using few resources beyond those needed for sample preparation and amplification. Combined with the quantitative and quality control capabilities of LRE, this kinetic-based approach has the potential to fundamentally transform how real-time qPCR is conducted.

## Introduction

Since its commercial introduction well over a decade ago, real-time quantitative PCR (qPCR) has come to play a prominent role in the life sciences, providing the foundation for a plethora of applications in basic research, pathogen detection and biomedical diagnostics. Nevertheless, a number of limitations associated with current methods have prevented the full potential of real-time qPCR from being realized. Paramount is the difficulty of implementing absolute quantification due to the necessity of constructing target-specific standard curves [1]. Not only has this impeded broad adoption of absolute quantification, reliance on standard curves makes absolute quantification impractical for large-scale applications.

**Figure 1:**
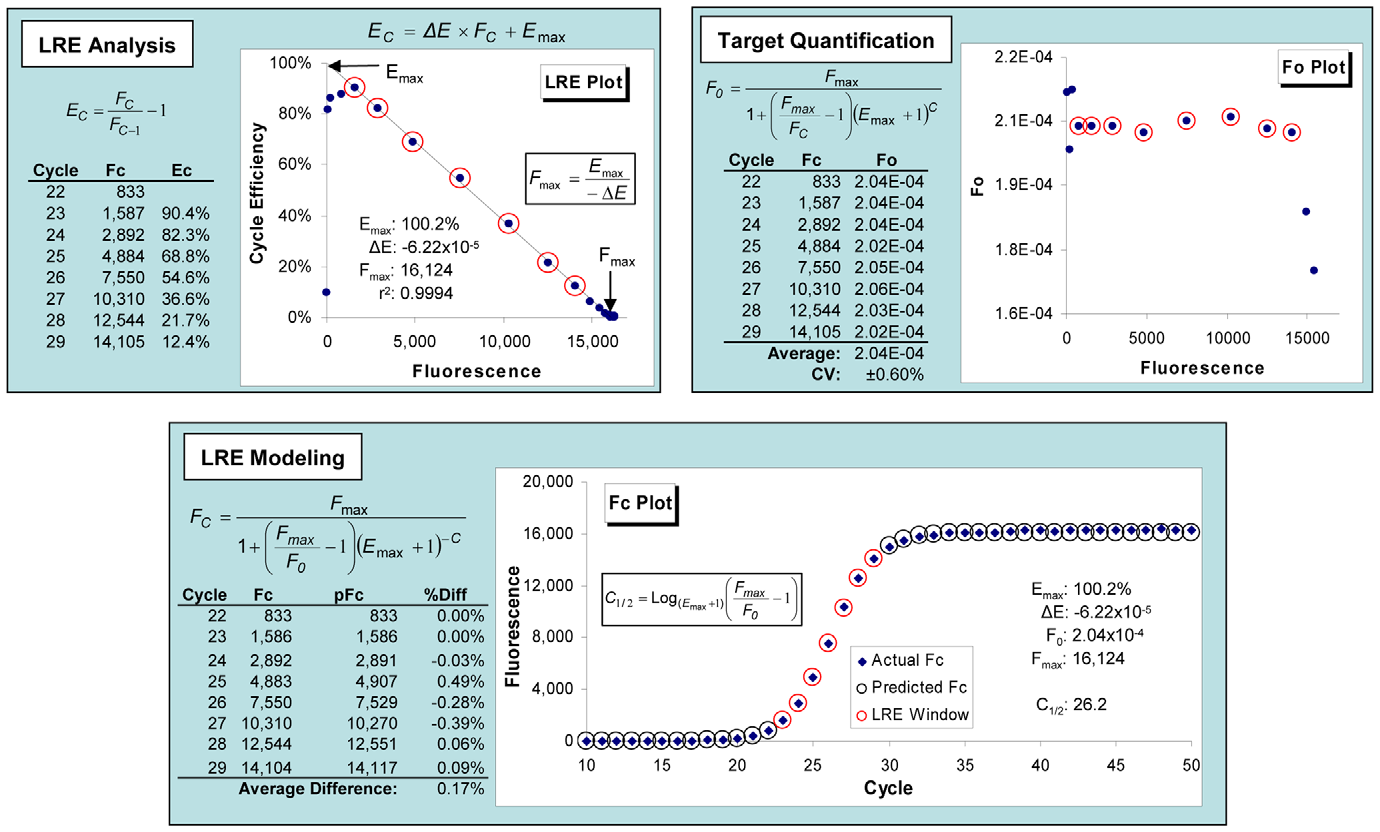
Overview of LRE-based real-time qPCR. As described in detail in a previous study [2], LRE originated from recognition that amplification efficiency is linearly coupled to amplicon quantity and is made up of three broad components. **LRE analysis** is implemented by first estimating cycle efficiency (E_C_) from the relative increase in reaction fluorescence over each individual thermocycle. E_C_ is then plotted against reaction fluorescence (F_C_) to produce the LRE plot. In addition to generating a linear representation of PCR amplification, linear regression analysis of the cycles within the central region of the profile (denoted by red circles and referred to as the “LRE window”) provides values for two kinetic parameters, the maximal amplification efficiency, E_max_ (Y intercept) and the rate of loss in amplification efficiency, ΔE (slope). **Target quantification** involves conversion of the F_C_ readings within the LRE window into target quantity (F_0_) using an equation derived from the classic Boltzmann sigmoid function. As described in a later section, the universal nature of SYBR Green I fluorescence allows the average F_0_ to be converted into the number of target molecules using an optical calibration factor (OCF). **LRE modeling** utilizes a second sigmoid function to calculate predicted F_C_ values for each thermocycle (pFc). Comparison of predicted to actual F_C_ within the LRE window typically produces an average difference of <0.5%, indicative of the remarkable precision that can be achieved. Profile position can be defined by C_1/2_, which is the fractional cycle at which reaction fluorescence reaches half of F_max_.

Based upon kinetic analysis of the fluorescence readings within the central region of an amplification profile, LRE provides an alternative methodology for conducting real-time qPCR (Figure 1). In addition to imparting exceptional quality control capabilities, the ability to exploit bacteriophage lambda genomic DNA (lambda gDNA) as a universal quantitative standard provides a simple and reliable approach to implementing absolute quantification. Furthermore, the ability to fully automate LRE analysis presents the prospect of developing high-throughput applications for absolute quantification that require few resources beyond those needed for target amplification [2].

The primary objective of this study was to evaluate the performance capabilities of LRE, as well as assess the universal nature of SYBR Green I fluorescence. In addition to illustrating the ability of LRE to model PCR amplification under a variety of assay conditions, investigating the dynamic range of LRE quantification demonstrates that quantitative accuracy can be maintained down to a single target molecule. Of a more fundamental nature is the demonstration that Poisson distribution can explain apparent quantitative aberrancies observed for target quantities below ten molecules.

## Results

### Modeling PCR amplification

The dynamics of amplification efficiency are what most greatly distinguish LRE from the widely accepted exponential model of PCR amplification. Under an exponential model, amplification efficiency is constant, a presumption that contradicts the apparent loss of amplification efficiency that occurs within a real-time PCR profile. Nevertheless, the presence of a log-linear region, also referred to as the exponential phase, has long been presumed to be indicative of constant amplification efficiency within the lower region of an amplification profile [3], [4], [5], [6]. As we have previously reported, LRE modeling contests this interpretation, predicting instead that the presence of a log-linear region is a result of an exponential loss in amplification efficiency [7]. The fact that LRE refutes the exponential character that has historically been ascribed to real-time PCR, could challenge the ability of LRE to accurately model PCR amplification. It was therefore of interest to investigate the role of the two kinetic parameters predicted by the LRE model to govern PCR amplification, ΔE and E_max_.

One of the fundamental principles of the LRE model (Figure 1) is that the height of an amplification profile (F_max_) is dependent on ΔE and E_max_ as dictated by the equation:



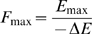
(1)


**Figure 2:**
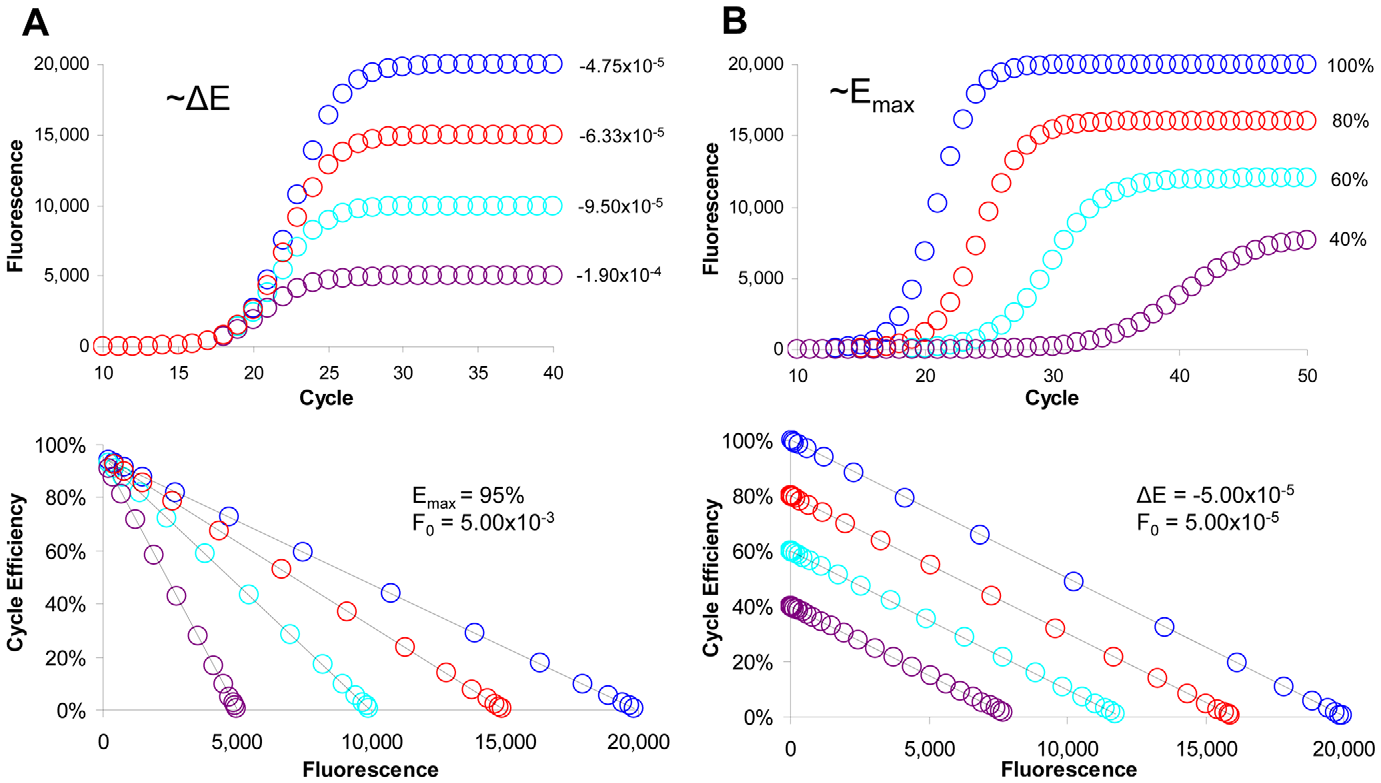
Mathematical modeling of PCR amplification. In order to examine the role of ΔE and E_max_, a series of mathematically generated amplification profiles were constructed using the LRE model. (**A**) Amplification profiles in which ΔE is progressively reduced, with the corresponding LRE plots presented in the lower panel. This illustrates the prediction that F_max_ is dependent on ΔE as described by equation 1. (**B**) Amplification profiles in which E_max_ is progressive reduced, with the corresponding LRE plots presented in the lower panel. This predicts that in addition to reducing F_max_, a reduction in E_max_ will also change the shape and position of a profile.

This predicts that decreasing ΔE will increase profile height, whereas decreasing E_max_ will reduce it. Mathematical modeling further predicts that decreasing E_max_ also impacts both the shape and position of an amplification profile, as illustrated in Figure 2.

**Figure 3:**
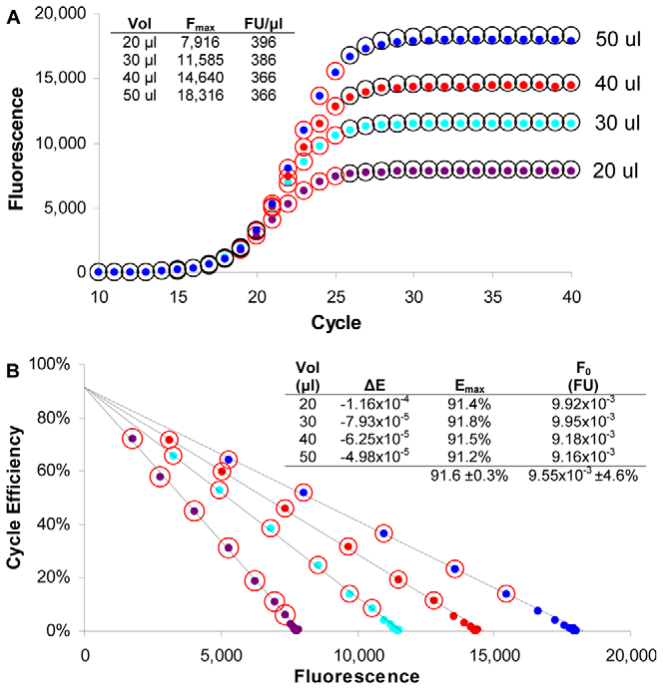
Impact of reaction volume on the ability of LRE to model PCR amplification. Equal quantities of lambda gDNA were amplified in PCR reactions containing progressively greater volumes. (**A**) F_C_ plots demonstrating that profile height is not only dependent on reaction volume, but that the concentration of amplicon DNA in the plateau phase (FU/µl) is similar for all reaction volumes. Predicted reaction fluorescence (circles) closely correlate with the actual fluorescence readings (dots). (**B**) LRE plots of the corresponding profiles demonstrates that increasing reaction volume has no impact on E_max_ (Y intercept), but does produce a proportional increase in F_max_ (X intercept). The numerical inlay provides a summary of the LRE analysis, which generated very similar F_0_ values. The cycles included within the LRE window are designated by red circles. Vol, reaction volume; FU/µl, fluorescence units per µl of reaction at F_max_.

To empirically test these predictions, a series of amplification reactions were conducted, the first of which involved reducing ΔE by progressively increasing reaction volume. Consistent with the mathematical predictions, profile height was found to be linearly dependent on reaction volume (Figure 3). This further revealed that similar concentrations of amplicon DNA were produced in the plateau phase (i.e. FU/µl), regardless of the profile height. Profile height has also been found to be independent of amplicon size (data not shown). Taken together, this suggests that cessation of PCR amplification involves a saturation mechanism that is coupled with amplicon mass. Very similar F_0_ values were also generated, indicating that LRE quantification is not dependent on reaction volume.

**Figure 4 pone-0009731-g004:**
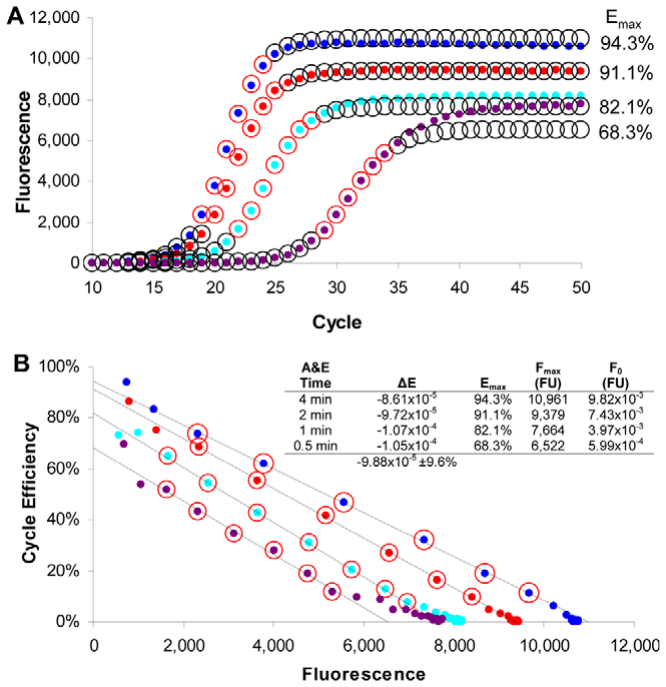
Impact of amplification efficiency on the ability of LRE to model PCR amplification. Equal quantities of lambda gDNA were amplified in which the time of annealing and elongation (A&E) was progressively reduced over four consecutive runs. (**A**) F_C_ plots reveal a progressive reduction in F_max_ along with changes in profile position and shape as E_max_ was reduced. The predicted reaction fluorescence (circles) correlate well with the actual fluorescence readings (dots), further corroborating the ability of LRE to model PCR amplification. Nevertheless, it should be noted that loss of conformity within the plateau phase (referred to as plateau drift) is apparent at the two shortest A&E times, a trend that has been observed under other suboptimal assay conditions (data not shown). (**B**) LRE plots reveal little difference in ΔE (slope), with a progressive loss in F_max_ (X intercept) as E_max_ (Y intercept) is reduced, a trend that is very similar to the mathematical predictions shown in Figure 2B. The cycles included within the LRE window are designated by red circles.

To examine the impact of reducing amplification efficiency, a second series of amplification runs were conducted in which the time of annealing and elongation (A&E) were progressively reduced, which also demonstrated a close correlation to the mathematical predictions of the LRE model (Figure 4). Nevertheless, this dataset reveals a loss of conformity within the plateau phase at the two lowest A&E times, a phenomenon that we have found can be diagnostic of suboptimal amplification conditions. Although not addressed in this study, this suggests that loss of conformity to the LRE model could be used to identify aberrant amplification kinetics that can impact assay performance (data not shown). Another important aspect revealed by this dataset is a progressive underestimation of target quantity as E_max_ is reduced, as reflected by the predicted F_0_ (Figure 4). While this could be related to inaccuracies of LRE modeling, it is more likely due to a disproportional loss in the efficiency of primer annealing and elongation to lambda gDNA molecules (referred to as target priming efficiency), in comparison with amplicon priming efficiency from which E_max_ is derived (see [2] for additional details concerning target priming efficiency). These datasets thus confirm the ability of LRE to model PCR amplification with remarkable precision.

### Optical calibration

**Figure 5:**
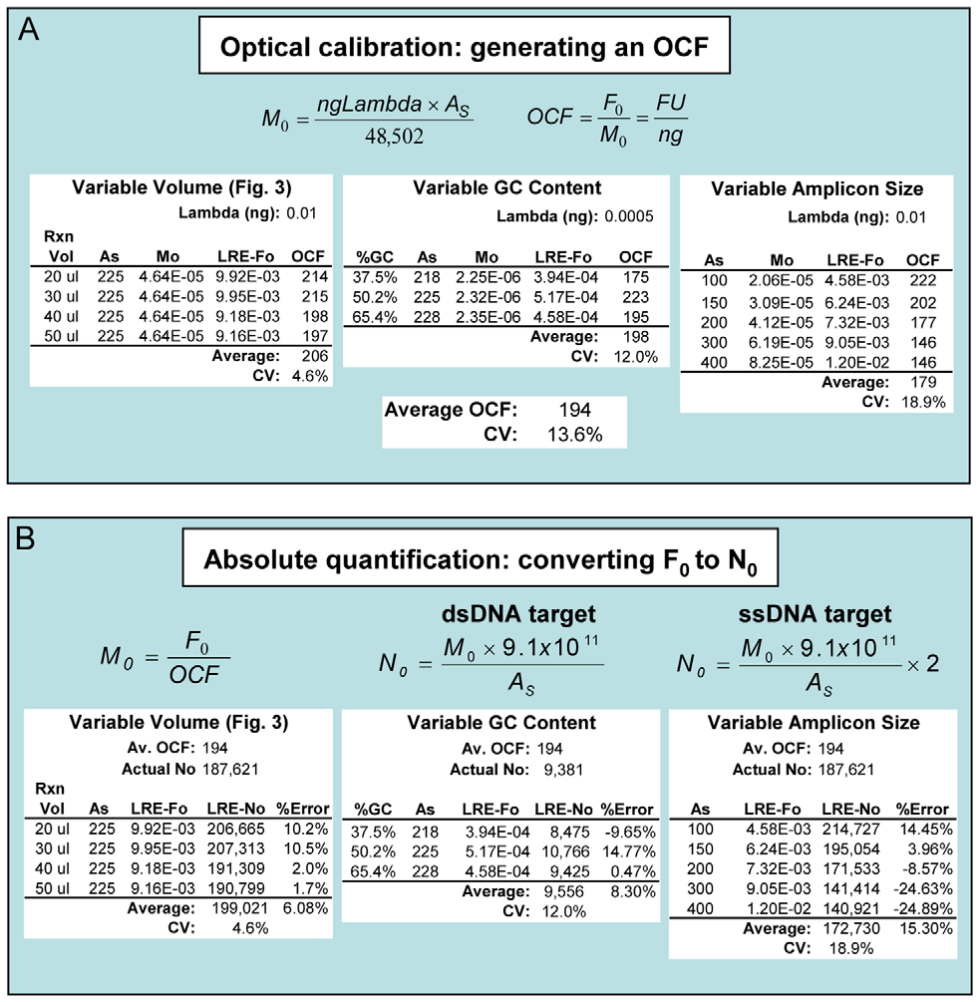
Absolute quantification via optical calibration. Three datasets are presented, demonstrating that the fluorescence intensity generated by SYBR Green I is independent of reaction volume, GC content and amplicon size. Note that the mathematics of optical calibration is described in more detail in a previous study [2]. (**A**) A known quantity of lambda gDNA is first amplified, from which an average F_0_ value is determined (LRE-Fo). An optical calibration factor (OCF) is then calculated by dividing the average F_0_ by the mass of the amplicon region derived from the lambda gDNA added to the reaction (M_0_). OCF is thus expressed as fluorescence units per nanogram of dsDNA (FU/ng). For lambda gDNA, M_0_ is calculated by multiplying the nanograms of lambda gDNA that are amplified by the amplicon size (A_S_), and dividing by the genome size of lambda (48,502 bp). Overall, the similarity of the respective OCF values indicates that any difference in fluorescence intensity generated by these eight lambda amplicons is small. (**B**) To determine the number of target molecules within a sample (N_0_), the process is reversed. M_0_ is first calculated by dividing F_0_ by an average OCF. N_0_ is then calculated by multiplying M_0_ by the number of base pairs per nanogram of dsDNA (9.1×10^11^ bp/ng) and dividing by the amplicon size (A_s_). For single-stranded DNA targets, such as cDNA, N_0_ must be doubled as the OCF is derived from a double-stranded standard. The level of accuracy that can be achieved using an average OCF is illustrated by taking the F_0_ values from A and comparing their respective N_0_ values to the predicted number of target molecules, expressed here as percent error. Note that the reaction volume dataset that were taken from Figure 3 was produced using the same reaction setup and instrument used for the GC content and amplicon size datasets.

Originating from recognition that standard curves derive absolute scale from the mass of amplicon DNA at threshold [1], optical calibration was first implemented during early attempts to apply sigmoidal modeling to real-time qPCR [8], which was further refined during development and testing of LRE [2]. Based on converting target quantity from fluorescence units (F_0_) to the number of target molecules (N_0_), the central premise of optical calibration is that absolute scale can be established using a single quantitative standard, if it can be assumed that all amplicons generate similar fluorescence intensities. It was therefore of interest to determine if GC content or amplicon size impacts SYBR Green I fluorescence intensity (i.e. FU/bp). In addition to its utility as a quantitative standard, the large genome of lambda allowed selection of amplicons with extreme GC contents. In combination with a series of amplicons that range in size from 100–400 bp (i.e. reflective of amplicons used in real-time qPCR), LRE analysis demonstrated that neither GC content nor amplicon size had any substantive impact on fluorescence intensity (Figure 5).

**Figure 6:**
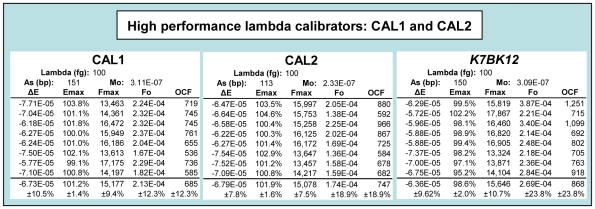
Comparison of three lambda amplicons used for optical calibration. Excel summaries of the optical calibrations taken from eight runs conducted over a 4-month period, with data from each individual run presented across each respective row. Although K7BK12 has performed well in previous studies, extensive testing has shown that CAL1 and CAL2 are exceptionally reliable, reflected in part by generating E_max_ standard deviations near ±1.5%. A_s_, amplicon size; M_0_, mass of the amplicon region within the target expressed in nanograms.

These datasets also illustrate the utility of lambda gDNA for performance testing. For example, the variance in OCF values provides an indication of the run-to-run reproducibility that can be achieved with LRE, which is typically less than ±20% for lambda calibrations. Consistent with this low variance, the application of an average OCF for conversion of F_0_ values into the number of target molecules (N_0_) further demonstrates the high level of quantitative accuracy that can be achieved with LRE (Figure 5B). Moreover, Figure 6 presents optical calibrations produced by two high-performance lambda amplicons conducted over a 4-month period, which further demonstrates that quantitative variances of less than ±20% can routinely be achieved with LRE. Note also that a similar range of quantitative variances was generated during LRE quantification of eleven cDNA targets [2].

### Dynamic range

**Figure 7:**
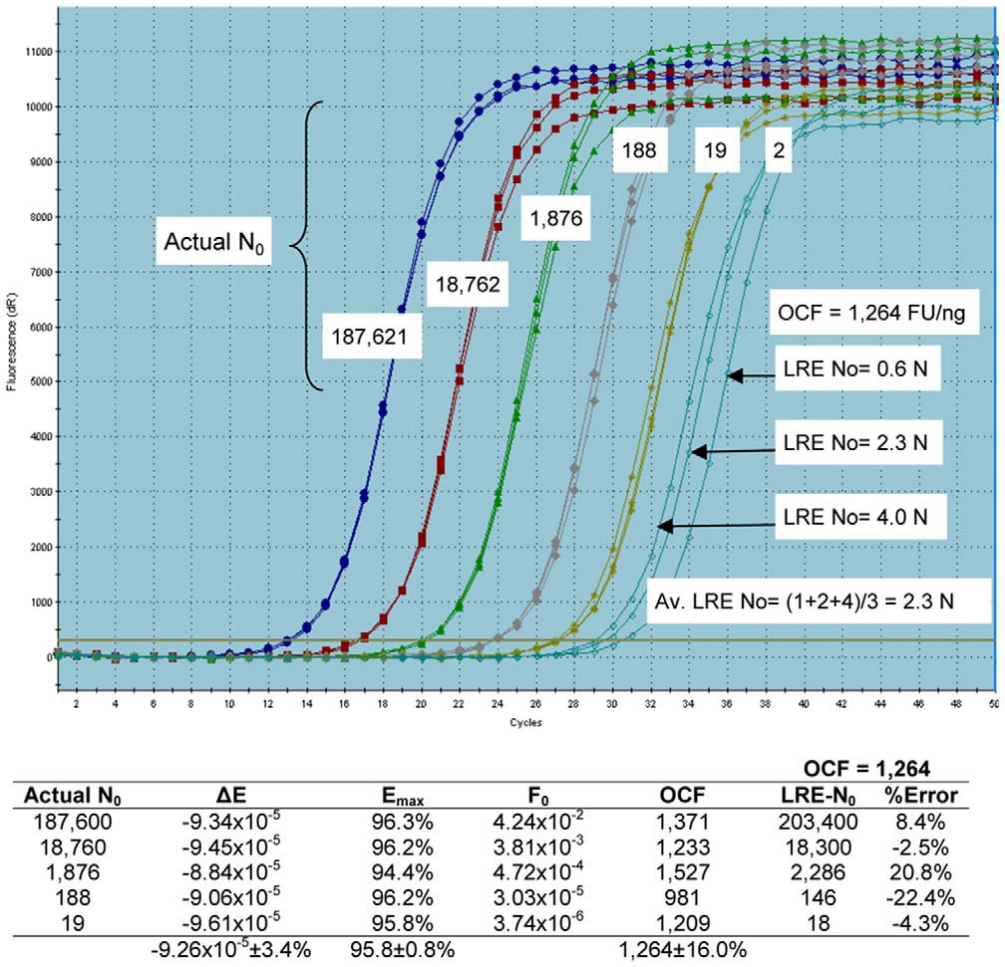
Amplification of serially diluted lambda gDNA generates profile scattering at low target quantities. Triplicate replicate amplifications of six quantities of lambda gDNA produce tight profile clustering, except for the two-molecule sample. Profile scattering implies loss of quantitative efficacy, a presumption supported by LRE quantification that generates target quantities that range from 1–4 molecules. Nevertheless, these LRE-based quantities produce an average of 2.3 molecules, which is close to the predicted target quantity. The numerical inlay summarizes the LRE analysis from which an average optical calibration factor (OCF) was derived.

Historically, the dynamic range of a real-time qPCR assay has been defined by serial dilution of a target-specific standard [9]. An example of this approach is presented in Figure 7, which demonstrates that replicate profiles become scattered when the target quantity is below ten molecules. Although profile scattering suggests that the lower limit of quantitative capability has been reached, averaging the LRE-derived quantities of the two molecule samples produces a value close to the predicted quantity, challenging such a simple interpretation.

**Figure 8:**
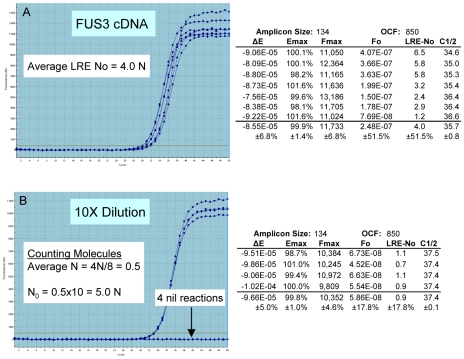
Implications of approaching limiting dilution. (**A**) Eight replicate amplifications of a low-abundance transcript produced extensive profile scattering, which could be interpreted as a catastrophic loss of quantitative accuracy. LRE quantification, as summarized in the numerical inlay, supports such a contention, generating N_0_ values ranging from 1–6 molecules, with a CV of ±51.5%. Nevertheless, an alternative explanation is provided by Poisson distribution, which dictates that large variations in target quantity occur between aliquots taken from samples containing a very low target concentration. (**B**) Diluting the sample 10X restores profile clustering for four of the eight replicate reactions, with LRE quantification predicting that these profiles originated from the amplification of a single target molecule. The production of nil reactions is predicted to be a result of aliquots lacking any target molecules, which is characteristic of reaching limiting dilution. Confirmation of absolute accuracy is provided by “counting” the total number of target molecules and dividing by the total number of reactions, which predicts that the target concentration in the undiluted sample is 5.0 molecules per aliquot. Furthermore, the tight clustering of single molecule profiles provides a striking example of the precision that can be achieved with PCR amplification, which in turn underpins the high level of quantitative accuracy that can be achieved with real-time qPCR.

**Figure 9:**
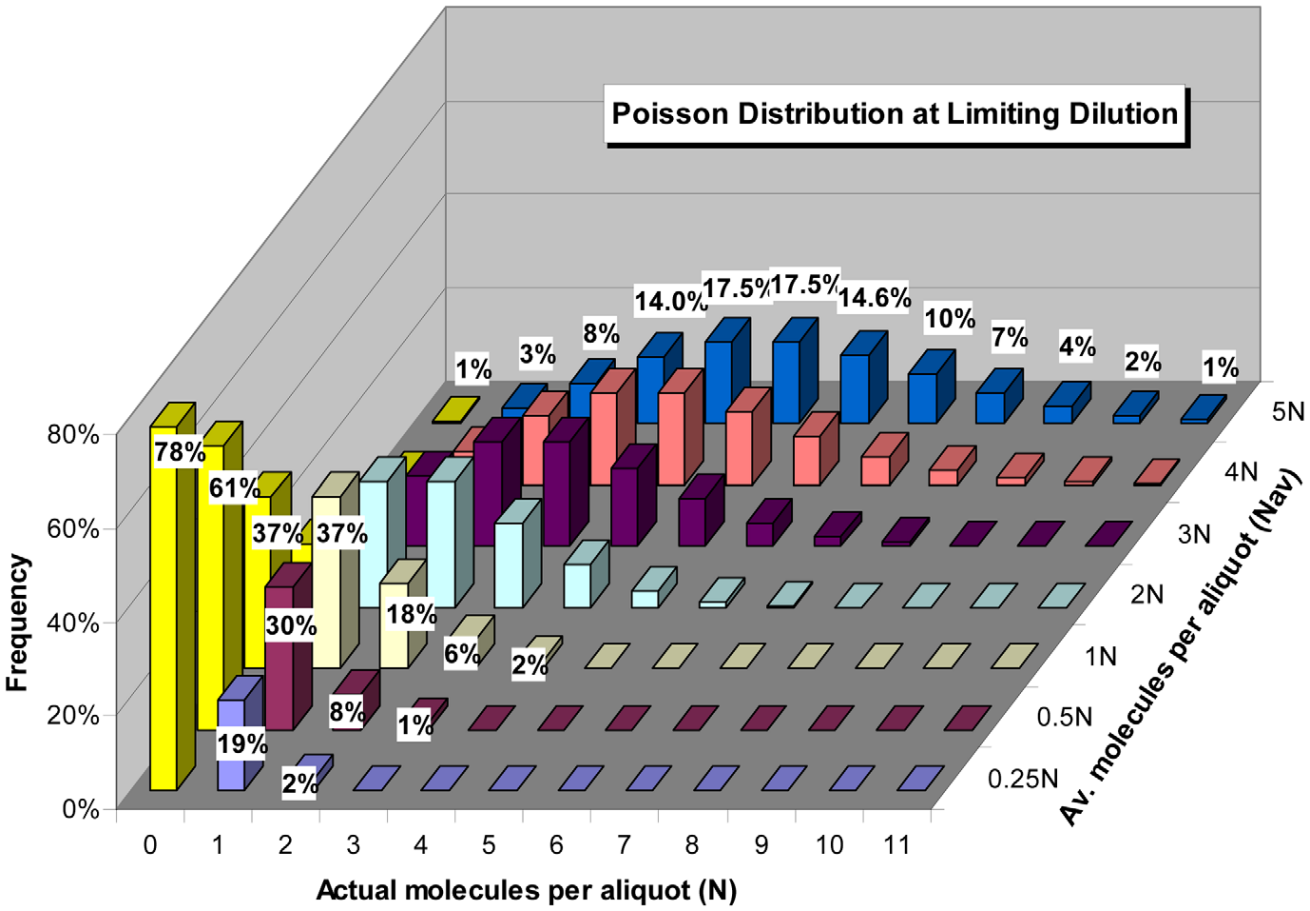
Poisson distribution of target molecules at very low concentrations. Poisson distribution dictates that aliquots taken at low target concentrations will contain a range of target quantities, such that a specific quantity of target (X-axis, N) is produced at a frequency (Y-axis, percent of all aliquots) that is dependent on target concentration (Z-axis, N_av_). Zero molecule aliquots (0 N) are designated by yellow bars, which in the absence of non-specific amplification will fail to produce an amplification profile.

Figure 8 provides an alternative interpretation for the dynamic range of real-time qPCR, which is that the lower limit of quantification is defined not by an innate limitation of qPCR, but rather by reaching “limiting dilution”. Indeed, it is a relatively simple matter to provide explanations for both profile scattering and the production of nil reactions by applying Poisson distribution. Sometimes called the “law of small numbers”, Poisson distribution describes the probability of rare independent events in relation to the average frequency of the event. When applied to real-time qPCR, the event represents the number of target molecules contained within an individual aliquot, with the average represented by the target concentration, and the rarity of the event represented by very low target concentration. As such, Poisson distribution provides the ability to predict the proportion of aliquots that will contain a specific number of target molecules based on target concentration (N_av_), as illustrated in Figure 9.

Referred to as “Poisson noise”, it is variability in the actual target quantity within individual aliquots that produces the scattering of replicate profiles. For example, in the case of a target concentration of 5 N_av_, only 17.5% of all aliquots will actually contain 5 target molecules, with the remaining aliquots predicted to contain quantities ranging roughly from 2 to 8 target molecules. In terms of real-time qPCR, this would be predicted to produce amplification profiles scattered over a two cycle region (i.e. 4-fold range of target quantity), which is indeed what is observed in Figure 8A. Furthermore, the frequency of aliquots that contain no target molecules becomes significant as the target concentration falls below two molecules per aliquot, consistent with that observed in Figure 8B. Importantly, Poisson noise has no relationship to the quantitative accuracy generated by individual amplification reactions. As addressed further below, an effective estimate of target quantity can be achieved by averaging the N_0_ values produced by replicate amplifications, although this average must include nil reactions (i.e. 0 N), as illustrated in Figure 8B.

Even though averaging of replicate determinations can be effective in overcoming Poisson noise, Wang and Spadoro [10] describe in detail how to conduct absolute quantification through the application of Poisson distribution, not to those reactions that produce a profile but rather to the frequency of nil reactions using the equation:
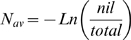
(2)where *nil* is the number of reactions that fail to produce an amplification profile and *total* is the total number of replicate reactions conducted. This allows absolute quantification to be conducted when target concentration is low enough to generate nil reactions. Note that conducting absolute quantification in this fashion is independent of the optical and kinetics parameters of real-time qPCR, and thus can be applied to any qPCR assay, irrespective of enzymology, detection chemistry or instrumentation.

**Figure 10:**
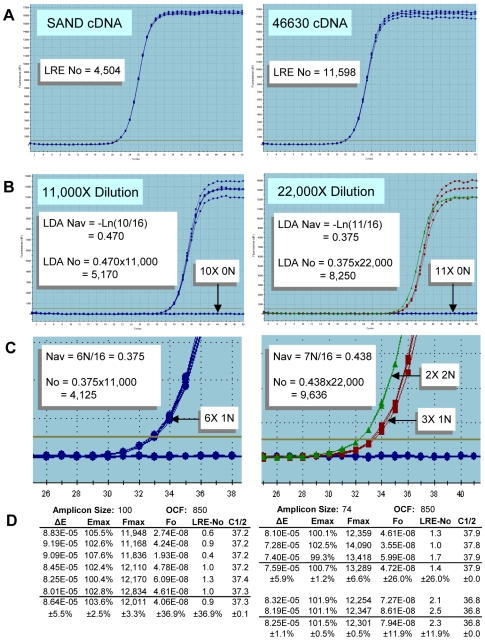
Verification of quantitative accuracy. (**A**) Two cDNA targets were quantified using LRE (N_0_  =  transcripts per 10 ng of total RNA), based on four replicate amplification reactions. The tight clustering of the replicate profiles is reflective of the high level of precision generated by these amplicons. Note, however, that for unknown reasons RNase H treatment has been found to dramatically disrupt replicate profile clustering for some amplicons (data not shown). (**B**) Samples were diluted to a predicted target concentration of about 0.5 N per aliquot and 16 replicate aliquots amplified. Similar to Figure 8B, a high level of profile clustering was generated, including putative 1 N and 2 N clusters for the 46630 target. The LDA quantifications, which rely solely on the frequency of nil reactions, correlate well with the LRE-based quantifications. (**C**) Counting target molecules as illustrated in Figure 8B correlates well with both LRE and LDA quantification, supporting the contention that these clusters were produced by amplification of one or two target molecules. (**D**) Summaries of the LRE analysis of the 1 N and 2 N profiles further demonstrate the ability of LRE to maintain quantitative accuracy down to a single target molecule. Note also the precise one cycle separation between the putative 1 N and 2 N clusters from the 46630 target, which is consistent with a one-fold difference in target quantity.

Initially implemented during development and testing of LRE [2], absolute quantification can be achieved for any sample by diluting the target below one molecule per aliquot, a method we refer to as “limiting dilution assay” or LDA. Thus, once an initial estimate of target quantity has been determined using real-time qPCR, a sample is diluted near to a predicted 0.5 N per aliquot and 16–48 replicate amplification reactions conducted (the more replicates, the greater the resolution [10]; however, as few as 16 replicates have been found to provide a reasonably reliable estimate). Two examples of this approach are presented in Figure 10, which further illustrates the quantitative capabilities of LRE.

One of the key attributes of LDA is that it is self validating. If the sample is underdiluted, no nil reactions will be produced, whereas overdiluting the sample will not generate any amplification profiles. The only major qualification is that nonspecific amplification products (e.g. primer dimers) are either absent or can be identified, which for SYBR Green I-based assays can be accomplished via melting curves. LDA is simple to conduct, does not require an external quantitative standard, and has in practice proven to be very reliable.

## Discussion

### Developing a new perspective for real-time qPCR

Based on defining the relative position of an amplification profile, all commercial real-time qPCR platforms currently rely on analysis of profile position. Despite the relative simplicity and inveterate nature of positional analysis, it is apparent that a number of innate limitations can compromise both the utility and efficacy of real-time qPCR. Foremost is the fact that positional analysis does not directly provide information about amplification efficiency, which is a major determinant of profile position. Determination of amplification efficiency is thus essential to achieving reliable quantification [11], [12].

Another shortcoming of positional analysis is that most methods depend on selection of a fluorescence threshold (F_t_) to generate the single point that defines profile position (e.g. C_t_). As such, any variation in F_t_ selection will generate inconsistencies, such that a one-fold difference in F_t_ generates about a one-fold difference in the apparent quantity of a target. Accurate comparison of profile position thus requires that F_t_ be fixed to a single value [1], a fact that is frequently overlooked.

The vagaries of positional analysis are further compounded for SYBR Green I-based assays in that profile position is dependent on amplicon size, another fact that is generally unrecognized. Assuming identical amplification efficiencies and target quantity, a one-fold increase in amplicon size will produce an amplification profile that is one cycle earlier, which roughly corresponds to a one-fold increase in the apparent target quantity. Exacerbated by the large number of available choices for detection chemistry, enzyme formulation and instrumentation, combined with a paucity of performance benchmarks, it is not surprising that a large number of studies have expressed concerns about the general efficacy of real-time qPCR [13], [14], [15], [16], [17], [18], [19], [20], [21], [22], [23].

Work with LRE in combination with LDA provided an alternative perspective that contested many of the supposed limitations of real-time qPCR. A notable example are the single molecule quantifications presented in Figures 8 and 10, which demonstrate that real-time qPCR can be a remarkably accurate technology. This contention has been corroborated through performance testing using lambda gDNA as a quantitative standard, which has generated many datasets illustrating the exceptional quantitative capabilities of real-time qPCR (e.g. Figures 5, 6 and 7; also see [7]; data not shown). This work also illustrated the outstanding utility of absolute quantification. Foremost is the ability to transcend details of assay design and implementation, such that quantitative data generated by disparate qPCR platforms can be directly compared. This is best exemplified by the platform independency of LDA, that in combination with the ease of implementation and inherent self-validation, provides a reliable, universally applicable method for independent verification of quantitative accuracy [2], [24]. This in turn facilitates the establishment of performance standards based on absolute accuracy that should, among other things, help address concerns about the efficacy of real-time qPCR.

Other methods that play an important role in verifying quantitative accuracy include comparing target quantities generated by multiple amplicons, which has been found to be effective in detecting base pair mismatches between a primer and the target (e.g. single nucleotide polymorphs) that can generate unrecognized, potentially large quantitative errors (see [2] for additional details; see also [25]). Similar to its utility as a universal quantitative standard for standard curve analysis [16], genomic DNA isolated from the target species has also proven to be effective not only for testing quantitative precision across multiple amplicons and targets, but can also be useful for assessing target specificity (data not shown).

### Exploiting the universal nature of SYBR Green I fluorescence

One of the seminal elements of the sigmoidal modeling from which LRE was derived [2], [8], [26] is that target quantity is expressed in fluorescence units (F_0_). This is similar in many ways to DNA quantification using a dedicated fluorometer and a fluorescence dye such as Pico Green, in which DNA mass is determined by comparing the fluorescence intensity produced by a sample, to that generated by a quantitative standard, such as lambda gDNA. Optical calibration of a real-time PCR reaction is based on the same principle, except that the target fluorescence is determined indirectly using real-time qPCR. An important caveat, however, is the implicit assumption that all amplicons generate similar fluorescence intensities (i.e. FU/bp). Demonstration that SYBR Green I fluorescence intensity generated during real-time PCR is not impacted by GC content or amplicon size (Figure 5) supports this contention. Note that Spandidos et al. have also reported SYBR Green I fluorescence to be independent of GC content and amplicon size, based on analysis of purified amplicon DNA [27].

The universality of SYBR Green I fluorescence thus presents the prospect of standardizing absolute quantification by shifting reliance from target-specific standards to a single, well-defined universal standard. Another important attribute of adopting a universal standard is the quality control capabilities it provides. For instance, in addition to allowing inter-run performance to be monitored, it provides a performance benchmark useful for assessing differences in enzyme formulation, cycling regime, instrumentation and/or data processing methodologies. This presents the prospect of adopting performance standards that, for example, could be based on CAL1 and/or CAL2 (Figure 6).

### The utility of absolute qPCR for expression profiling

Although absolute quantification can provide a biological perspective essential to many applications, such as for biomedical diagnostics involving viral load and residual disease, it is the application of absolute quantification to gene expression profiling that could have some of the most profound implications. This is primarily due to the quantitative perspective provided by absolute quantification, which could allow transcript quantities generated by any gene to be directly compared with any other gene, within and between any number of samples. A prominent example comes from an early real-time qPCR study in which 29 yeast transcripts were profiled [28]. Notably, this study has two parallels with LRE-based quantification. The first is the use of a single universal quantitative standard (HIV RNA) for establishing absolute scale. The second was the ability to generate an average quantitative precision of ±20%, which is similar to what can be achieved with LRE. Interestingly, this was accomplished despite the fact that the analysis was conducted using ethidium bromide detection and a Perkin Elmer 9600 thermal cycler retrofitted with a handheld UV lamp. Comparison with SAGE quantification further extended the quantitative context of the study, allowing quantities to be expressed as the number of transcripts per cell. This revealed that real-time qPCR was about two magnitudes more sensitive than that provided by their SAGE dataset, allowing transcript quantities as low as 0.00075 copies per cell to be measured.

This study was later expanded to 275 transcripts that included 185 transcription factors, which demonstrated that transcript quantity in yeast varies over six orders of magnitude [29]. This study also revealed that microarray quantification based on raw fluorescence maintained correlation with the real-time qPCR quantifications down to about two copies per cell, and failed to effectively quantify nearly 50% of these transcripts. Czechowski et al. report similar results for real-time qPCR profiling of over 1400 Arabidopsis transcriptional factors, in which less than 55% were detectable using the 22K Arabidopsis Affymetrix array [30]. While this large proportion of undetectable transcripts is likely biased by the low level of expression characteristic of transcriptional factors, it is nevertheless disconcerting that such a large number of transcripts falls below the apparent detection limit of microarray analysis [31].

Based on the exquisite sensitivity and resolution provided by real-time qPCR, LRE could fulfill an important niche for large-scale gene expression profiling. This is particularly true for studies that require analysis of large numbers of samples, such as for temporal sampling or tissue/population surveys, which are not well suited for massively parallel technologies such as microarrays, SAGE, MPSS or next-generation sequencing (reviewed in [32]), due primarily to their high cost and technical complexity. In contrast, LRE does not require any specialized materials or equipment beyond that required to conduct a standard SYBR Green I assay, although this assumes that a user has access to both the raw fluorescence readings and a computer program for LRE analysis. In an attempt to address this latter need, a Java API is currently under development, which extends the Java applet developed earlier for automated LRE analysis [2], with the expectation that it will provide a foundation for developing the software needed to conduct large-scale absolute quantification based on LRE.

## Methods

A detailed description of reaction setup, fluorescence data acquisition, and LRE data processing methodologies has been provided previously [2]. All the datasets in this study were generated with the QuantiTect SYBR Green I enzyme formulation (Qiagen). Table 1 provides a listing of all the primer sets used in this study. Lambda gDNA was obtained from New England BioLabs and diluted to the specified quantities with 10 mM Tris using siliconized microfuge tubes.

**Table 1:** Amplicon primer sequences.

Figure	Amplicon	5′ Primer	3′ Primer
Lambda gDNA			
Figure 1, 6	CAL1	AGACGAATGCCAGGTCATCTGAAACAG	CTTTTGCTCTGCGATGCTGATACCG
Figure 6	CAL2	GTATCCATCGGGTGTGCTTCCTGATATG	GTGGGTGTGCGACTTAATTCCATCCT
Figure 6, 7	K7BK12 (150 bp)	CTGCTGGCCGGAACTAATGAATTTATTGGT	ATGCCACGATGCCTCATCACTGTTG
Figure 3, 4, 5	K7K8 (55.2% GC)	CTGCTGGCCGGAACTAATGAATTTATTGGTGAAGGT	ACCGAGTTCAGAAATAAATAACGCGTCGCCGGAA
Figure 5	K3K4 (37.6% GC)	TGGCCTGCTGTCCCTGTTATGGAGTAATCGTTTT	CCATATTTCATGCGTTCAGTCTTAAAAGCAATTGGCGGTGAT
	K5K6 (65.4% GC)	AGCTTGGGATCAGCAGCCTGACGGAT	GGGCGATAATGCCGTTGTAACCGGTCAT
	K7BK10 (100 bp)	CTGCTGGCCGGAACTAATGAATTTATTGGT	TATCTGGCGGTGCAATATCGGTACTGTT
	K9K10 (200 bp)	CGGAAAACACCGTCAAAAACATTGCATTTAACTATATTGTG	TATCTGGCGGTGCAATATCGGTACTGTT
	K11K10 (300 bp)	ATTGGTGCGCACCAGCATCCG	TATCTGGCGGTGCAATATCGGTACTGTT
	K13K10 (400 b bp)	GGGTATTGCTTATTTATCGAAAACGGACAGTCAG	TATCTGGCGGTGCAATATCGGTACTGTT
cDNA			
Figure 8	FUS-K3K4	CTCTTACCTCCAACGATACTCCTCTCGATT	CTAGTAGAAGTCATCGAGAGAGATATTCTCAACGG
Figure 10	SAND-K3K4	TGCATGTAAAAGGATTGGGACCCCACAAG	GCTGCATAGAGTTCAAAATCTGGTGTGACCC
	46630-K1K4	AGTTCGTTTTCTCAAGGTTTGGGAGAAGAGCG	CCGCTTTCGTTATGTATCGAACCCACTCGA

The datasets presented in Figures 3–5 were generated during the early stages of LRE development, and were conducted with a MX3000P thermocycler (Stratagene) using a 25 µl reaction volume and a cycling regime consisting of 95°C for 15 min for enzyme activation, followed by 40 cycles of 95°C, 10 s and 68°C 3 min, with 5 replicate optical readings taken at the end of each cycle. All the other datasets were conducted on a different MX3000P instrument, with 3 replicate optical readings taken at the end of each cycle. The calibration datasets presented in Figures 1 and 6 used a reaction volume of 10 µl, with a cycling regime consisting of 95°C for 15 min for enzyme activation, followed by 50 cycles of 95°C, 10 s and 65°C, 2 min. The datasets presented in Figures 7 and 8 used a reaction volume of 5 µl containing 1 µl of sample, and used a cycling regime consisting of 95°C for 15 min for enzyme activation, followed by 50 cycles of 95°C, 10 s and 65°C, 3 min. The dataset presented in Figure 10 used a reaction volume of 5 µl containing 1 µl of the sample and used a cycling regime consisting of 95°C for 15 min for enzyme activation, followed by 50 cycles of 95°C, 10 s and 65°C, 2 min.

The reverse transcription reactions used in Figures 8 and 10 were prepared as previously described [2]. It should be noted, however, that a RNase H treatment was not conducted, as it has been found to produce extensive scattering of replicate profiles for some cDNA targets (data not shown). It should also be noted that we have found that many commercial enzyme formulations contain significant amounts of lambda gDNA, which can cofound the ability to generate the low target concentrations presented in Figure 7.
